# Green Synthesis of ZnO Nanoparticles From *Spirulina platensis*: Antimicrobial and Cytotoxic Evaluation With Molecular Docking Studies

**DOI:** 10.1002/open.202500515

**Published:** 2025-12-09

**Authors:** Muhammad Rizwan, Izaz Khan, Syed Sikandar Shah, Amir hamza khan, Abdurahman Hajinur Hirad, Saira Naz, Arshad Farid, Taimoor Khan, Said Hassan

**Affiliations:** ^1^ Center for Biotechnology and Microbiology University of Swat Swat Pakistan; ^2^ Department of Clinical Pharmacy and Pharmacology RAK College of Pharmacy RAK Medical and Health Sciences University Ras Al KHaimah United Arab Emirates; ^3^ Institute of Biotechnology and Genetic Engineering The University of Agriculture Peshawar Pakistan; ^4^ Department of Botany and Microbiology College of Science King Saud University Riyadh Saudi Arabia; ^5^ Department of Chemistry Faculty of Physical and Applied Sciences University of Haripur Haripur Pakistan; ^6^ Gomal Center of Biochemistry and Biotechnology Gomal University D.I.Khan Pakistan; ^7^ School of Life sciences University of Warwick Coventry UK; ^8^ Department of Health and Biological Sciences Abasyn University Peshawar Pakistan

**Keywords:** antibacterial, antibiofilm, cytotoxicity, insilico study, zinc oxide nanoparticles

## Abstract

Zinc oxide nanoparticles (ZnO NPs) have drawn some attention due to its antimicrobial properties and potential for their usage in biomedicine. ZnO NPs were synthesized using algal biomass filtrate, characterized subsequently, and the antimicrobial, antibiofilm, and cytotoxic activities of the ZnO NPs were evaluated. The synthesized ZnO NPs were characterized with fourier transform infrared, X‐ray Diffraction, scanning electron microscopic, and transmission electron microscopy to analyze their morphological and compositional properties. Agar well diffusion, time‐kill assays and antibiofilm experiments were conducted to assess antibacterial efficacy, while *Artemia salina nauplii* were used to evaluate cytotoxicity. It was found that ZnO NPs had hexagonal wurtzite crystal structure, uniform nanoscale morphology ranging 20–90 nm, and high phase purity. ZnO NPs exhibited potent antibacterial activity, further confirmed by molecular docking analysis. Strong antibiofilm efficacy, reducing biofilm biomass by up to 95%. Exceptional results were shown by wet lab and docking validation. The PBP2a protein of *S. aureus* showed a binding score of −7.7, indicating strong inhibition by the ZnO NPs. These nanoparticles exhibit a dose‐dependent response, where minimal toxicity was observed at lower concentrations. This study highlights the potential of algae‐mediated ZnO NPs as a green, sustainable antimicrobial platform that may mitigate the microbial resistance problem.

## Graphical abstract

Algal‐extract‐derived ZnO nanoparticles show shape anisotropy and are bio capped by phytochemicals. These green‐synthesized NPs strongly inhibit S. aureus and K. pneumoniae biofilms. Molecular docking suggests stable binding of surface biomolecules to microbial targets, highlighting synergistic antibacterial mechanisms.

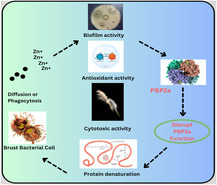

## 1 Introduction

Nanotechnology, the science of manipulating materials at the nanoscale, has emerged as a powerful tool in various fields due to the remarkable characteristics exhibited by nanoparticles [1]. These distinctive properties arise from the superfine surface area, quantum behavior, and bio‐chemical compatibility of nanomaterials. Particles with at least one dimension within the 1–100 nm range are called nanoparticles. They have found application in various fields and many industries; from medicine to environmental science. Over the years, NPs have significant contribution in biomedical scientific research, that is, site‐specific drug delivery, diagnostic assays, and imaging techniques. Besides, they enhance other properties like mechanical strength, electrical conductivity ad other properties of materials used in industries. Nanoparticles have potent applications in environment remediation, waste‐water treatment, hydrocarbon removal, and energy harvesting [2, 3]. Conventional methods of NPs synthesis generally include high temperature, huge energy consumption, and toxic chemicals, which are harmful to human health and to the environment as well. This has led to green synthetic approaches using plants, microorganisms, and algae. Additionally, in green synthesis the same natural biomolecules act as reducing and stabilizing molecule, thus eliminating hazardous chemicals. These environmental friendly strategies are green chemistry approaches represents the biocompatible and environmentally benign routes for nanoparticles fabrication [4].

In this study, ZnO NPs were synthesized with the help of biological extract of *Spirulina platensis* acting as a natural reductant [5]. The as‐synthesized ZnO NPs exhibit enhanced antimicrobial and photocatalytic activities, attributed to the proteins and polysaccharides present in the natural extract, which is stabilizing agent for this stationary phase of NPs [6]. Here the filamentous cyanobacterium *Nostoc muscorum* used for ZnO NPs biosynthesis has been responsible for the formation of nanoparticles with hexagonal crystalline morphology and efficient antimicrobial activities [2]. ZnO nanoparticles derived from *Spirulina* have also been reported in photocatalysis, especially in the decolorization of dyes in wastewater treatment. Similarly, ZnO derived from Mg–Al layer has shown enhanced photocatalytic efficacy for the degradation of methylene blue under UV light [7, 8].

ZnO NPs are well known for their broad‐spectrum antimicrobial efficiency, targeting all types of microorganisms, starting from Gram‐positive and Gram‐negative bacteria, fungi, up to viruses. The antimicrobial ability of nanoparticles is attributed to their intrinsic physicochemical characteristics like, large surface to volume ratio, an effective large reactive surface, and the ability to generate reactive oxygen species (ROS). All these properties collectively contribute to strong microbial inhibition, thus making ZnO NPs a good candidate for replacing traditional antibiotics, especially in case of increasing risks of resistive bacterial strains.

Beside the antimicrobial activity, ZnO NPs exhibit synergistic effects when combined with conventional antibiotics. For instance, a synergistic interaction between ZnO NPs and antibiotics such as ciprofloxacin and ceftazidime was reported against multidrug‐resistant (MDR) *A. baumannii* [9]. This enhanced bacterial inhibition was attributed to the increased uptake of antibiotics by ZnO NPs [10]. The combined effect could serve the purpose of reducing the required antibiotic dose thereby lowering side effects and slowing down the development of resistive bacterial strains. ZnO NPs have strong antibacterial activity against food born bacteria like *Salmonella typhimurium*, *Staphylococcus aureus* making them worthy of being used as food preservative [11].

ZnO NPs have also shown notable antioxidant properties. It has been found that ZnO NPs synthesized *via* both aqueous and polyol routes exhibited high levels of free radical scavenging ability [12] ZnO can effectively neutralize DPPH and super oxide radicals. This antioxidant capacity of nanoparticles was found to be dose dependent, which is maximal at higher nanoparticle concentrations [13]. Synthesizing ZnO NPs using green synthetic methods has turned out favorable for achieving a higher antioxidant activity of the resultant nanoparticles. Furthermore, plant derived biomolecules were utilized in this study as capping and bioactivity stabilizing the ZnO NPs as hypothesized by [14]. Following the same pattern, *Polygala tenuifolia* root extract was used for ZnO NPs synthesis and established substantive free radical scavenging properties and anti‐inflammatory activity [15].

ZnO NPs have also emerged as promising drug delivery agents, particularly in cancer therapy. ZnO NPs can be effectively used to deliver cytotoxic chemotherapeutic agents, thus enhancing therapeutic precision [16]. ZnO NPs have facilitated the delivery of anticancer agents such as doxorubicin to get high and effective therapeutic benefits, reducing the side effects of the drug if administrated directly. The ability to release drugs at the optimal pH in the acidic tumor proliferation microcosol, as well as the ability to self‐assemble further highlights the potency of ZnO NPs as a versatile and intelligent nanocarrier for controlled and site‐specific cancer therapy [17].

Molecular docking (MD) technology is employed to evaluate the bioactivity of any recognized molecule or any synthesized compound through its interaction with target proteins. The evaluation of compound activity is performed by calculating binding affinities derived from molecular dynamics interactions, as outlined in various published scientific articles focusing on natural compounds from plant or microbial origins, as well as nanoparticles that function as antimicrobial or anticancer agents [18]. Also Molecular docking, a well‐recognized and versatile in silico method, was employed to evaluate the binding interactions between ZnO NPs, selected protein, and the chosen ligands [19]. The aim of this scientific investigation was to synthesize ZnO NPs utilizing a green synthetic approach and assess its effectiveness against specific multidrug‐resistant bacteria and other relevant proteins through molecular docking studies, in addition to its biomedical uses via diverse assays.

## 2 Material and Methods

### 2.1 Algal Growth and Storage

Fresh Cyanophyte (blue‐green algae) species were gathered from District Swat, Khyber Pakhtunkhwa, Pakistan, and stored in sealed sterile plastic bags. The algae was kept at low temperatures until they were grown on a freshly prepared growth medium to prevent deterioration. Additionally, water was used as the culture medium, which was rich in nutrients, to develop Cyanophyte (blue‐green algae). In addition, the algae were gently dried after extraction from the medium to remove any remaining moisture. After growth, the algae were harvested and washed with water to remove impurities, followed by shade drying to preserve the phenolic compounds that might degrade in direct sunlight or mechanical drying. The dry material was ground to get a fine powder and stored in air‐tight container for further experiments. Before experiments, the algal biomass was filtered using a Whatman filter paper to ensure purity [20].

### 2.2 Formulation of ZnO‐NPs

**FIGURE 1:**
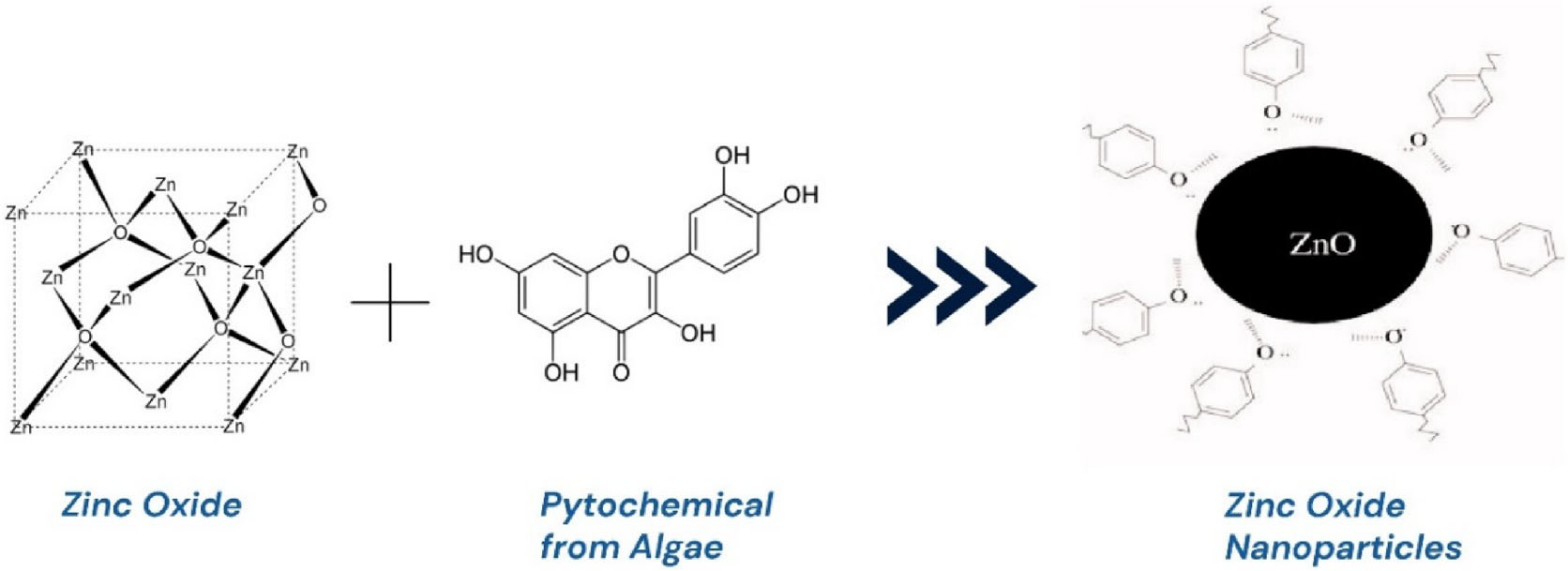
Proposed phytochemical‐mediated synthesis mechanism of ZnO nanoparticles.

The biomass of the microalga was used throughout the phase of logarithmic expansion. After centrifuging, any remaining media components were removed by rinsing the biomass three times with double‐distilled water that had been deionized. To get the biomass filtrate (supernatant), 100 milliliters of distilled water were mixed with 15 g of cleaned biomass and centrifuged again for 48 h. ZnO NPs were synthesized by dissolving 0.44 g of Zn (CH_3_COO)_2_·2H_2_O in 2 mL of distilled water, then adding 98 mL of the biomass filtrate to achieve a 2 mM last concentration. For a whole day, the mixture was shaken at 150 rpm and incubated at 30°C ± 2°C. To produce ZnO NPs powder. Proposed reaction mechanism between the zinc precursor and algal phytocompounds during green synthesis of ZnO nanoparticles (Figure 1). The resulting precipitate, which was white, gathered and oven‐dried for 24 h at 200°C [21].

### 2.3 Characterization of ZnO NPs

#### 2.3.1 FT‐IR Examination of Targeted ZnO NPs

Fourier transform infrared (FTIR) analysis was used to identify functional groups for the Cyanophyte (blue green algae) extract and the synthesized ZnO NPs. The analysis was performed to understand the role of these functional groups in the reduction of zinc ions and in the stabilization of the synthesized nanoparticles. Next, prepared the Cyanophyte sample and studied the functional groups in the extract to determine their part in nanoparticle synthesis. To prepare the ZnO NPs sample for FTIR analysis, ≈0.2 g of powdered ZnO NPs were thoroughly mixed with potassium bromide (KBr) in a 1:100 for uniformity. After that, the mixture was subjected to the high pressure, at which point a clear KBr disc is made that is used as the medium for the FTIR analysis. Spectra were taken for FTIR, from 400 to 4000 at 4.0 cm^−1^ resolution. The resulting spectra were analyzed on characteristic peaks of the different functional groups. By comparing the peaks of Cyanophyte extract with those of ZnO NPs, functional groups responsible for the reduction of the Zn ion and stabilization of the nanoparticles were elucidated. FTIR analysis was used to elucidate chemical composition and surface chemistry of synthesized ZnO NPs and it was found that FTIR analysis shows specific functional groups contributing to the synthesis of ZnO NPs [22, 23].

#### 2.3.2 Scanning Electron Microscopic (SEM) Analysis

SEM spectra were used to characterize the morphological characteristics of the bio‐synthesized ZnO NPs, such as its structure, shape, size, and distribution. In the current research, JEOL (JSM‐5910, Japan) model of a SEM were used. The system delivers a resolving power of 2.3 nm to a maximum of 300,000x with practical range 30 kV suitable for solid material detailed analysis. At first the sample was dried to remove any residual water in it and then a small amount of sample was mounted onto an aluminum SEM stub using double side adhesive carbon tape followed by coating of a thin layer of gold using a sputter coater in vacuum conditions to improve sample conductivity and prevent charging during imaging. We then put the sample in the SEM chamber and imaged at high vacuum. The operating voltage was set to 30 kV to get best resolution and best contrast. Images were taken at different magnifications from the samples for analyzing the size, shape, and surface morphology of the resulted ZnO‐NPs. The nanoparticles were closely studied for their uniformity and if any agglomeration of nanoparticles is possible. Detail of the structural features of the bio‐synthesized ZnO NPs were obtained from the SEM analysis [24].

#### 2.3.3 Transmission Electron Microscopy (TEM) Analysis

TEM analysis was conducted to verifying the size, shape, and overall morphology of the synthesized ZnO NPs. The equipment consisted of a JEOL‐JEM 2100 Electron Microscope (Japan) having resolution up to 1.4 Å and an optical zoom up to 1,500,000x. For TEM analysis a freshly prepared nanoparticle suspension was taken. A single drop was carefully placed on a copper TEM grid with a carbon film to get uniform deposition of nanoparticles on the grid surface, the grid was allowed to be air dried at room temperature. The sample grid was mounted onto the TEM holder and inserted into the vacuum chamber of the microscope for imaging. To preserve the structural integrity of the nanoparticles during analysis, the accelerating voltage of the microscope was adjusted to achieve optimum imaging and contrast resolution. High resolution images were captured at various magnifications to evaluate the size, shape, and dispersion of the ZnO‐NPs. Special attention was given to assessing the dimensions of individual nanoparticles as well as their aggregations. The nanoscale characteristics of ZnO‐NPs were thoroughly investigated using TEM to ensure uniformity and structural features. This detailed analysis provided insights into the synthetic process of nanoparticles and their applicability in various biomedical and environmental applications [22].

#### 2.3.4 X‐Ray Diffraction Analysis

A JEOL X‐Ray Diffractometer Model JDX‐3532 from Japan was used for the X‐ray diffraction (XRD) examinations. It has an operating voltage range of 20–40 kV, a current capability of 2.5–30 mA, and it uses CuK*α* (wavelength = 1.5418 Å) X‐rays. The range of 2*θ* encompassed 0°–160°. For examination, drained nanoparticles material will be applied to a glass screen at a rate of 0.5°/min for 2 s, covering the 2*θ* range of 4°–90° [25]. Furthermore, applying the commonly used Scherrer's formula to analyze the line length of the highest point with greatest intensity, the mean diameter of the particles of ZnO NPs was ascertained from the pattern of the XRD [26, 27].

### 2.4 Biological Applications

#### 2.4.1 Antibacterial Assay

##### 2.4.1.1 Agar Well Diffusion Assay

Antibacterial activity of ZnO‐NPs against *Klebsiella pneumoniae*, *Escherichia coli*, and *Staphylococcus aureus* were evaluated by the agar well diffusion assay. All the bacterial strains were purchased from a certified culture collection and revived in Luria‐Bertani (LB) broth under shaking conditions at 37°C for 24 h for exponential growth. Bacterial suspension was standardized to turbidity equivalent to 0.5 McFarland standard (≈1 × 10^8^ CFU/mL) and used to inoculate Muller Hinton Agar plates. Aseptically, sterile MHA plates were prepared and allowed to solidify. To guarantee uniform bacterial growth each plate was inoculated evenly with the bacterial suspension with a sterile cotton swab. In agar, sterile borer was used to make wells with a diameter of about 6 mm. 100 µg/mL ZnO NPs concentrations were prepared in sterile distilled water, and 50 µL of each concentration was added into the relevant wells. Inoculated plates were incubated at 37°C for 24 h and zones of inhibition around the wells were measured with a digital caliper. In all, the diameter of the inhibition zones was recorded in millimeters [28, 29].

##### 2.4.1.2 Time Kill Assay of ZnO NPs

Three bacterial strains employed in this study were *K. pneumoniae*, *E. coli*, and *S. aureus*. Short term storage of these strains on nutrient agar slants at 4°C and longtime storage was achieved by maintaining these strains in 20% glycerol at −80°C. A 50 μL aliquot each of the freshly prepared colonies was streaked on nutrient agar plates and inoculated into Mueller Hinton broth (MHB) and incubated at 37°C at 120 rpm with shaking for 16–18 h. Bacterial cultures were standardized to an optical density (OD) of 0.1 at 600 nm (OD_600_), equivalent to ≈1 × 10^8^ CFU mL^−1^, by using a UV–visible spectrophotometer. For current experiments the standardized inoculum was diluted 10‐fold into sterile MHB to a final bacterial density of about 1 × 10^6^ CFU mL^−1^. A bacterial suspension was prepared, treated with ZnO NPs (1, 10, 100 μg/mL) following serial dilutions in sterile distilled water. These concentrations were selected based on preliminary studies. Before mixing, the ZnO NPs suspension was sonicated for 30 min to ensure a homogeneous dispersion of nanoparticles, in equal volume, with bacterial suspensions in 96 well microplates. Incubation temperature and duration of incubation were 37°C and 0, 2, 4, 6, and 24 h, as samples were incubated under static conditions, and aliquots were taken at time points of 0, 2, 4, 6, and 24 h. At every time point, 100 µL of the bacterial nanoparticle suspension was sampled and optical density (OD_600_) was measured using a UV–visible spectrophotometer in conjunction with sterile MHB as a blank. Bacterial cell concentration (OD) was plotted against time to determine time‐kill curves. The following controls were used: positive controls (ciprofloxacin 1 μg/mL), negative controls (bacterial suspensions without the addition of ZnO‐NPs) and vehicle controls (ZnO NPs dispersant alone without active nanoparticles) [29, 30].

##### 2.4.1.3 Antibiofilm Potential of ZnO‐NPs

ZnO‐NPs were assessed for biofilm inhibition and eradication potential against *K. pneumoniae*, *E. coli* and *S. aureus* using crystal violet assay. Dispersion of ZnO NPs was performed in sterile deionized water at a concentration of 1 mg/mL and sonicated for 30 min to have uniform dispersion. *K. pneumoniae*, *E. coli* and *S. aureus* strains were grown at 37°C for 16‐18 h in pure culture in Luria Bertani (LB) broth with OD_600_ nm adjusted to 0.1 (≈10^6^cfu/mL). For the biofilm inhibition assay, 200 µL bacterial suspension was pipetted in a 96 well microtiter plate containing 20 µL each of ZnO NPs solution at different concentrations (10, 20, 50 and 100 µg/mL). Positive controls (bacteria without nanoparticles) and negative controls (sterile LB broth) were used, the plate was incubated at 37°C for 24 h. For the biofilm eradication assay, preformed biofilms were first made by culturing bacterial suspensions for 24 h and followed by washing of the wells with PBS to remove nonadhered cells. Further incubation for 24 h was performed with the addition of ZnO NPs to the wells containing the biofilms. The biofilms were incubated, washed with PBS, then fixed in methanol and stained with 0.1% crystal violet in water for 15 min followed by a wash with sterile distilled water. Biofilm biomass was quantified by solubilizing the crystal violet stain with 33% acetic acid and measuring absorbance at 590 nm [30].

### 2.5 Protein Ligand Docking

3D structure of Zn ONP were generated using Avogadro software, and docking were performed using PyRx, using ligands like zinc nanoparticle with beta‐lactamase, and AcrB protein of *K. pneumonia*, PBP2a, and alpha hemolysin of *S. aureus* DNA gyrase and dihydrofolate reductase of *E. coli.* First protein ligand ZnO NPs were made using Avogadro software, then structure was stabilized and suitable force field were added to this structure, and also bonds were added to it. Then all the proteins used in this study were obtained from online databases. After this PyRx were used for docking, all the proteins and ligand were first converted into pdbqt and then each complex were added to Gridbox having AutoGrid dimensions, and then docking were started, at the end each complex produces specific binding energy and RMSD, only those complexes were selected that had low RMSD and high binding energies.

### 2.6 Antioxidant Activity of ZnO NPs

Antioxidant activity via DPPH assay in a microtiter plate, 100 µL freshly prepared DPPH solution, 0.1 mM in methanol, was added into each 96 well plate. Afterwards, 100 µL of ZnO NPs solution or ascorbic acid solution at 10–200 μg/mL was added in respective wells. The reaction was complete after incubating the plate in the dark at room temperature for 30 min and gently mixing. The absorbance of the cultures was read at 517 nm using a microplate reader after incubation. IC50 values were determined relative to the control for comparative analysis of antioxidant potential, and the percentage of DPPH radical scavenging activity was calculated.

### 2.7 Cytotoxic Effects of ZnO NPs

The cytotoxic effects of ZnO NPs on Artemia salina were assessed based on hatching success and mortality observed visually. Observed movement and survival rates of nauplii in response to varying ZnO NPs concentrations were observed with the naked eye under natural light conditions.

## 3 Results

### 3.1 Physiochemical Characterization

#### 3.1.1 FTIR Results

**FIGURE 2:**
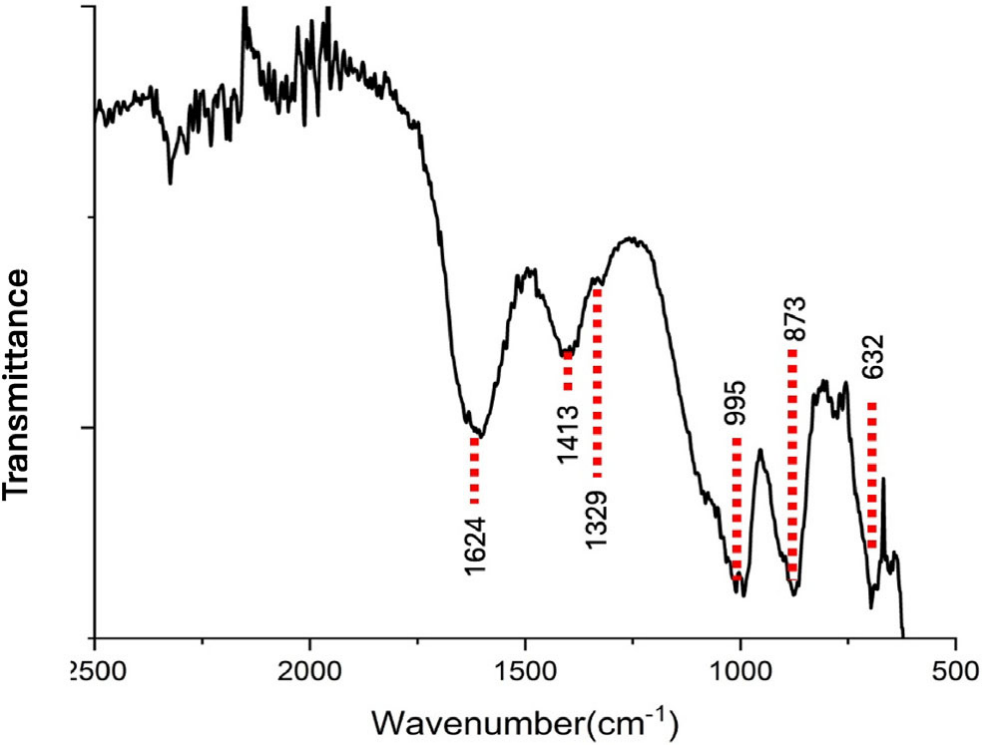
Representation of FTIR spectra of ZnO NPs, Various peaks represent different functional groups.

FTIR spectrum of ZnO nanoparticles shows key functional groups and lattice vibration for their structure and surface chemistry. Hydroxyl (O‐H) bending vibrations are reflected in the peak at 1624 cm^−1^, meaning surface adsorbed water, and peaks at 1413 and 1329 cm^−1^ are associated with carbonate or organic residues from synthesis. C–H bending or ZnO lattice interactions are likely involved in the band at 995 cm^−1^. In particular, the peak at 632 cm^−1^ corresponds to the Zn–O characteristic stretching vibration of the wurtzite crystal structure and strongly supports that the ZnO nanoparticles are well crystallized as shown in the Figure 2.

#### 3.1.2 XRD Analysis

**FIGURE 3:**
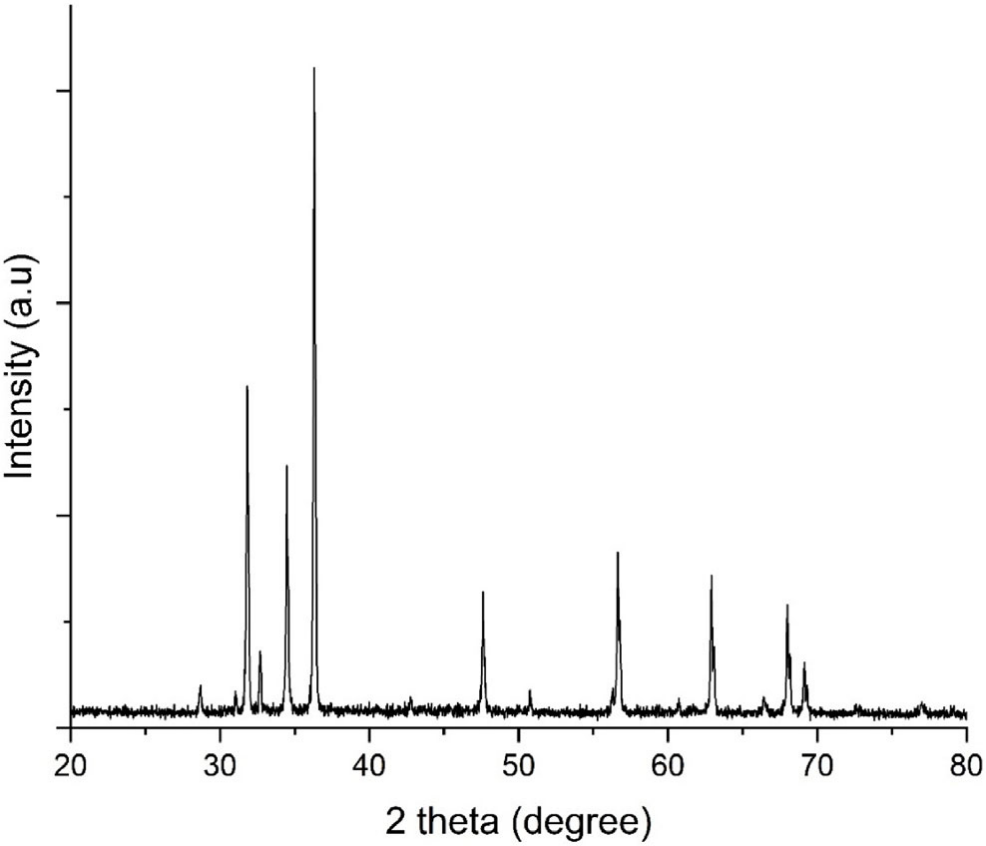
XRD pattern of ZnO NPs, diffraction Bragg peaks shown were 32, 33, 34, 37, 47, 57, 63, 68, and 69.

ZnO nanoparticles have shown the XRD pattern with well‐defined diffraction peaks of a hexagonal wurtzite crystal structure. Prominent peaks are observed at 2*θ* angles of 31.8°, 34.4°, 36.2°, 47.5°, 56.6°, 62.9°, and 67.9° indicating the crystalline nature of ZnO. The seven peaks are matched to (100), (002), (101), (102), (110), (103), and (112) crystallographic planes. These peaks are sharp and intense; indicative of good crystallinity, and the lack of extra peaks suggest phase purity of the sample, as shown in Figure 3.

#### 3.1.3 Scanning Electron Microscopy (SEM) Analysis

**FIGURE 4:**
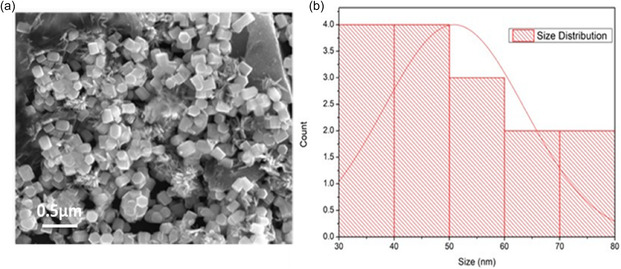
SEM histogram of ZnO NPs, shows most ZnONP size ranges from 40 to 50 nm.

The SEM image depicts the morphology of synthesized zinc oxide nanoparticles at a magnification of 30,000×. The histogram shows the nanoparticle size distribution, with the corresponding frequency (count) on the *y*‐axis and size intervals in nanometers (nm) on the *x*‐axis. The distribution shows that the highest bar indicates that most of the nanoparticles are sized in the 40–50 nm range. The distribution curve suggests a slightly right‐skewed distribution with a lower number of nanoparticles at larger size ranges. The particles range from a minimum of 35 to a maximum of 75 nm. While it is found that most nanoparticles belong to the size class of 40–50 nm, the presence of some larger‐sized nanoparticles contributes to greater dispersion that may even alter the overall sample homogeneity. The average size depicted through SEM was about 50 nm as shown in Figure 4.

#### 3.1.4 TEM

**FIGURE 5:**
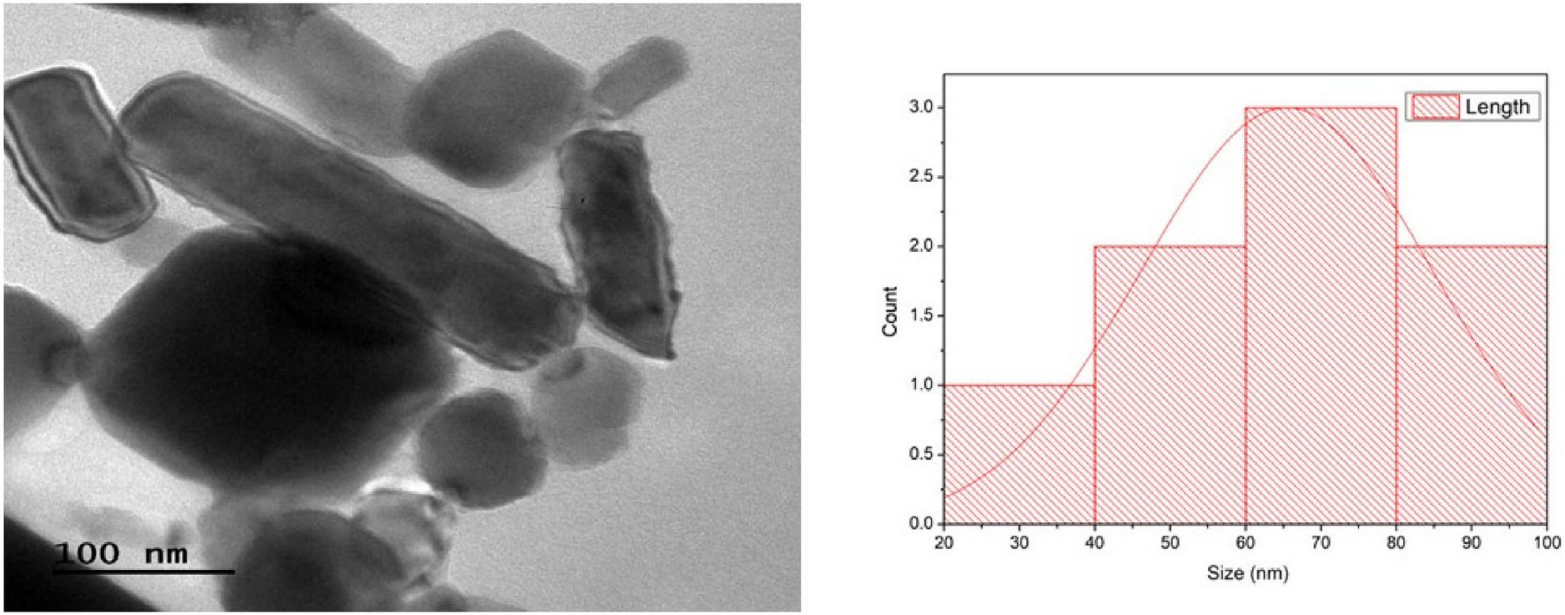
TEM image of ZnO nanoparticles showing clear shape anisotropy.

The histogram shows the nanoparticle size (nm) distribution across the size ranges on the *x*‐axis and counts on the *y*‐axis. The largest count in this bin indicates that the data implies most of the nanoparticles seems exhibiting a crystal‐clear anisotropy. The normal spread of the distribution suggests it may have a relatively wide size distribution, including particles as small as 37–94 nm. The fitted curve over the histogram shows a distribution nearly normal with the peak being up around the 60–70 nm range. So, the average size of the nanoparticles is 65 nm which is determined *via* image as shown in Figure 5.

### 3.2 Biological Applications

#### 3.2.1 Antibacterial Efficacy of ZnO‐NPs

Antibacterial efficacy of ZnO NPs against *E. coli*, *K. pneumoniae*, and *S. aureus* was superior to several conventional antibiotics. Furthermore, ZnO NPs exhibited an inhibition zone of 20 ± 1.54 mm against *E. coli*, which was much more promising compared to cefepime (14.6 ± 1.3 mm). Hence, ZnO NPs can potentially be used to disrupt bacterial growth more effectively than cefepime. Likewise, ZnO NPs revealed an inhibition zone of 24 ± 1.62 mm for *K. pneumoniae* which was prominently larger than ciprofloxacin (12.7 ± 0.9 mm) and amoxicillin‐clavulanic acid (13.6 ± 1.1 mm). This emphasizes the antimicrobial potential of ZnO‐NPs, which may be related to their ROS generating capacity and their ability to disturb bacterial membranes. Zone of inhibition of 19 ± 1.73 mm was recorded for ZnO NPs in *S. aureus*, significantly larger than that of ciprofloxacin (10.6 ± 0.4 mm), also comparable to vancomycin (13.4 ± 0.9 mm) as shown in Table 1.

**TABLE 1:** Inhibition zones of ZnO NPs and antibiotics against *E. coli*, *K. pneumoniae*, and *S. aureus*.

Bacterial strains	Antibiotics	Zone of inhibition of antibiotics	Zone of inhibition of ZnO NPs, mm
*E. coli*	Ciprofloxacin	11.5 ± 1.1	
	Imipenem	11.1 ± 1.0	
	Cefepime	14.6 ± 1.3	20 ± 1.54
	Amoxicillin+clavulanic acid	13.4 ± 0.8	
*K. pneumoniae*	Ciprofloxacin	12.7 ± 0.9	24 ± 1.62
	Imipenem	13.4 ± 1.0	
	Vancomycin	14.2 ± 0.7	
	Amoxicillin+clavulanic acid	13.6 ± 1.1	
*S.aureus*	Ciprofloxacin	10.6 ± 0.4	19 ± 1.73
	Imipenem	12.9 ± 1.1	
	Vancomycin	13.4 ± 0.9	
	Amoxicillin+clavulanic acid	12.2 ± 0.4	

#### 3.2.2 Time Kill Assay

**FIGURE 6:**
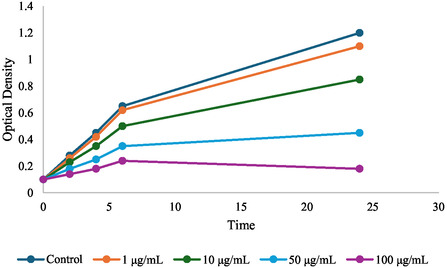
Time Kill Curve shows ZnO‐NPs had a significant antibacterial activity against *K. pneumoniae*, *E. coli*, and *S. aureus* by reduction in optical density (OD_600_).

Results showed that ZnO‐NPs had a significant antibacterial activity against *K. pneumoniae*, *E. coli*, and *S. aureus* by reduction in optical density (OD_600_) over time, as shown in the Figure 6. ZnO NPs exhibit antibacterial activity in a concentration‐dependent manner, with higher the concentrations of ZnO NPs (50–100 μg/mL), the more decrease of OD_600_ compared to control groups. Exposure to 100 μg/mL concentrations resulted in a statistically significant reduction in bacterial growth within 6 h of exposure, with the greatest inhibition occurring at 24 h (as measured by OD_600_). *E. coli* was especially sensitive, with OD_600_ values falling by 85% compared to the untreated control, whereas values for *K. pneumoniae* and *S. aureus* were reduced by ≈78% and 80%, respectively.

**FIGURE 8:**
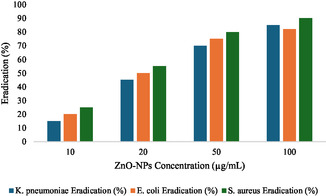
Biofilm eradication efficiency of ZnO NPs, shows *S. aureus* biofilms were found to be more sensitive to ZnO NPs than *K. pneumonia*.

Lower concentrations (1 μg/mL), OD_600_ values for ZnO NPs did not differ substantially from those for the negative control during 24 h incubation. Time‐kill curves generated from OD_600_ data indicated time‐dependent inhibition of bacterial growth when exposure concentrations of 50 μg/mL or above were used where significant bacterial density reduction was observed after 4–6 h of exposure. In contrast, bacterial cultures treated with ZnO NPs showed a less drastic decline in OD_600_ after 2 h because of the relative potency of the ZnO NPs compared to ciprofloxacin (positive control). Negative and vehicle controls displayed no significant OD_600_ change over incubation period and confirmed that the observed antibacterial effect was not due to the dispersant or any other external factors, as the ZnO‐NPs shown in Figure 6.

### 3.3 Antibiofilm Potential

#### 3.3.1 Biofilm Inhibition

**FIGURE 7:**
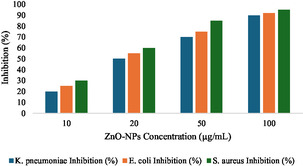
Biofilm Inhibition capacity of ZnO NPs, shows 95% inhibition at 100 µg/mL for *S. aureus* and slightly less sensitive 90% inhibition at 100 µg/mL.

Biofilm formation by the tested bacterial strains was effectively inhibited by ZnO‐NPs. When biofilm was treated with the lowest concentration (10 µg/mL), inhibition was moderate, lowering biofilm biomass by 20%–30% compared to the positive control. At 20 µg/mL, biofilm inhibition was significantly enhanced, up to about 50%–60%. At higher concentrations (50 and 100 µg/mL), there was significant biofilm inhibition (70–85 and 90–95% biomass reduction, respectively). The sensitivity was among the highest (95% inhibition at 100 µg/mL) for *S. aureus* and slightly less sensitive (90% inhibition at 100 µg/mL) to *K. pneumoniae* as shown in Figure 7.

#### 3.3.2 Biofilm Eradication

Preformed biofilms were removed by ZnO‐NPs, indicating their potential in biofilm eradication. The biofilm biomass was reduced from 15 to 25% across the strains at 10 µg/mL. The effect was concentration dependent and was 45%–55% at 20 µg/mL and 65%–75% at 50 µg/mL. The ZnO NPs eradicated 80%–90% preformed biofilm biomass at 100 µg/mL. As with biofilm eradication, *S. aureus* biofilms were found to be more sensitive to ZnO NPs than *K. pneumoniae* and *E. coli*, and a clearing rate of 90%, 85% and 82% was achieved at 100 µg/mL, respectively as shown in Figure 8.

### 3.4 Comparative Docking Analysis Across Multiple Pathogens

Microbial potential of ZnO NPs, a comparative docking analysis were conducted against six target protein of three different bacterial species *K. pneumonia*, *S. aureus*, and *E. coli*. The binding affinity were used to assess the strength between ZnO NPs and the target bacterial proteins.

**FIGURE 9:**
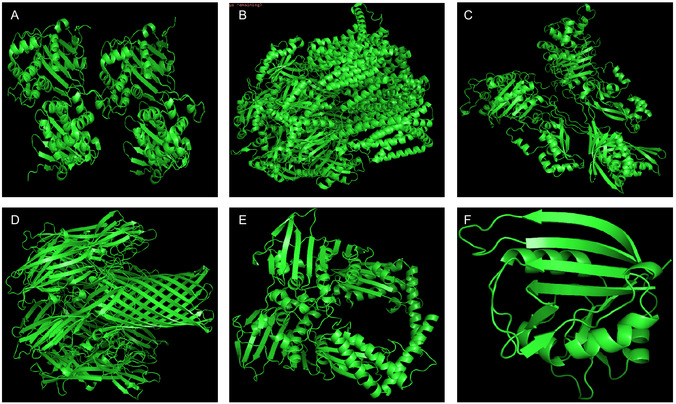
3D structure of Beta‐lactamase (a) AcrB. (b) PBP2a. (c) Alpha Hemolysin. (d) DNA gyrase. (e) Dihydrofolate reductase. (f) of *K. pneumoniae*, *S. aureus* and *E. coli*.

**FIGURE 11:**
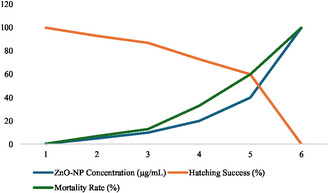
Cytotoxic effects of zinc oxide nanoparticles (ZnO‐NPs) on *Artemia salina*, also increasing ZnO NPs concentrations leading to reduced hatching success and increased nauplii mortality.

Among all the targets the strongest binding affinity toward PBP2a from *S. aureus* (−7.7 kcal/mol) followed by AcrB (−6.6 kcal/mol) and alpha‐hemolysin (−6.5 kcal/mol). The lowest binding affinity were observed against beta‐lactamase from *K. pneumonia* (−6.0 kcal/mol) and DNA gyrase from *E. coli* as shown in Table 2. These results indicate that *S. aureus* is the most susceptible bacteria to these nanoparticles insilico, consistent with the wet lab findings where its shows the greatest sensitivity to ZnO NPs shown in the Figure 11. Also 3D structure of Beta‐lactamase (A), AcrB (B), PBP2a (C), Alpha Hemolysin (D) and DNA gyrase (E), Dihydrofolate reductase (F) were shown in Figure 9.

**TABLE 2:** Binding affinities of ZnO NPs with target proteins of *E. coli*, *K. pneumoniae*, and *S. aureus*, indicating that *S. aureus* is the most susceptible bacteria.

Bacterial Species	Target Protein	Protein Function	Binding Affinity of ZnO NPs, Kcal/mol
*K. pneumoniae*	B‐lactamase	Antibiotic Resistance Enzyme	−6.0
*K. pneumoniae*	AcrB	Pump out antibiotics contributing to resistance	−6.6
*Staphylococcus aureus*	PBP2a	Cell wall synthesis (B‐lactam target)	−7.7
*Staphylococcus aureus*	Alpha‐hemolysin	Toxin (virulence factor)	−6.5
*Escherichia coli*	DNA Gyrase	DNA replication Enzyme	−6.1
*Escherichia coli*	Dihydrofolate Reductase	Folate biosynthesis enzyme	−6.3

Strong binding affinity of ZnO NPs to PBP2a from *S. aureus* is due to the enzyme key role in antibiotic resistance and the nanoparticles multifunctional antibacterial properties. PBP2a allows MRSA to maintain cell wall synthesis despite B lactam antibiotics, but ZnO NPs disrupt this by binding to the key residue in the enzyme active or allosteric site. The interaction is strengthened by ZnO ability to release Zn^2+^ ions generate ROS, and interact electrostatically with the bacterial surface. These likely to inhibit the transpeptidase activity of PBP2a impairing cell wall crosslinking and leading to bacterial death. The docking result showing the lowest binding energy (−7.7 kcal/mol) with PBP2a aligns with wet lab, where *S. aureus* showed the highest sensitivity towards ZnO.

### 3.5 Antioxidant Activity

**FIGURE 10:**
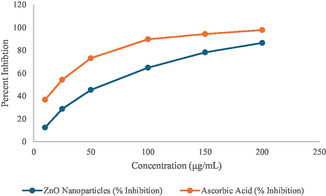
DPPH radical scavenging activity of ZnO nanoparticles, as shown that ZnO NPs concentration increased from 10 to 200 μg/mL, the percentage Inhibition values increased.

DPPH radical scavenging activity of ZnO nanoparticles was concentration dependent. As the ZnO nanoparticle concentration increased from 10 to 200 μg/mL, the percentage inhibition values increased. The IC50 value (concentration that inhibited 50% DPPH radicals) of ZnO nanoparticles was calculated to be 72.5 μg/mL whereas that of ascorbic acid (the standard antioxidant) being 21.3 μg/mL. This implies that though ZnO nanoparticles show fairly great antioxidant properties, their activity is less than that of ascorbic acid as shown in Figure 10.

### 3.6 Cytotoxicity

Cytotoxic effects of zinc oxide nanoparticles (ZnO‐NPs) on *Artemia salina* were assessed by observing hatching success and mortality rates visually, without the need for specialized equipment. The results revealed a clear dose‐dependent response, with increasing ZnO NPs concentrations leading to reduced hatching success and increased nauplii mortality. In the control group (0 µg/mL), all 15 eggs successfully hatched, and the nauplii remained active and mobile throughout the observation period, indicating 100% hatching success and 0% mortality. At 5 µg/mL, hatching success slightly decreased to 93%, with one nauplius exhibiting no movement, resulting in a mortality rate of 7%. Increasing the concentration to 10 µg/mL reduced hatching success further to 87%, with two nauplii succumbing, corresponding to a 13% mortality rate. At 20 µg/mL, hatching success dropped significantly to 73%, with five nauplii found immobile by the end of the exposure period, translating to a mortality rate of 33%. At 40 µg/mL, the adverse effects became more pronounced, with only 60% of the eggs hatching and 9 nauplii dying, resulting in a 60% mortality rate. The highest tested concentration (100 µg/mL) entirely inhibited hatching, with none of the eggs hatching and all nauplii immobile, leading to a 100% mortality rate. These results highlight the dose‐dependent toxicity of ZnO NPs on *Artemia salina*, with higher nanoparticle concentrations severely impairing hatching success and causing complete nauplii mortality at 100 µg/mL as shown in the Figure 11.

**FIGURE 12:**
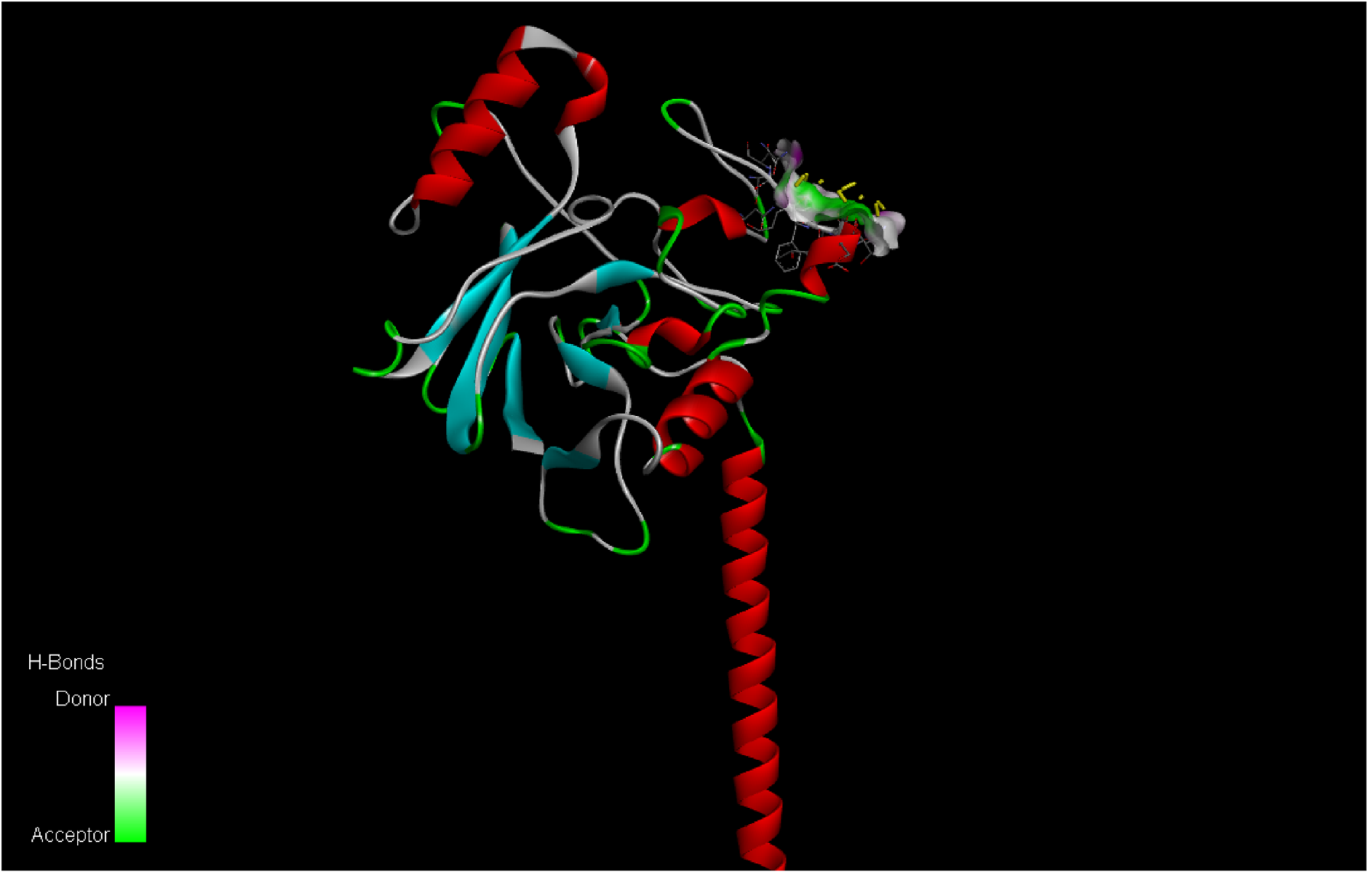
Zinc oxide nanoparticles (ZnO‐NPs) ligand bounded to Na+,K+‐ATPase alpha2 single chain protein of *Artemia salina* with a docking score of −5.1.

**FIGURE 13:**
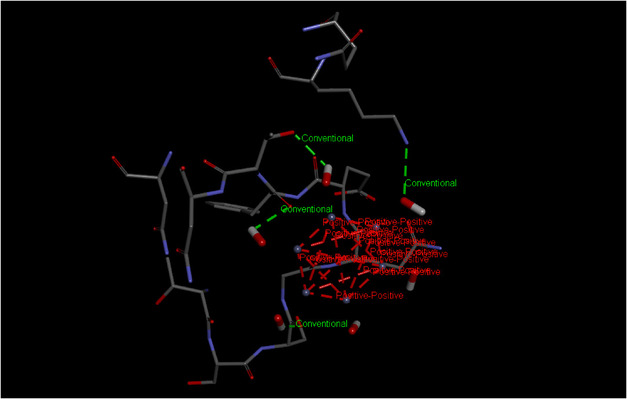
Zinc oxide nanoparticles (ZnO‐NPs) ligand bounded to Na+,K+‐ATPase alpha2 single chain protein of *Artemia salina* with 4 conventional bonding and many positive–positive interactions, generated by Discovery studio.

Figures 12 and 13, the ligand ZnO‐NPs form complex with Na+,K+‐ATPase alpha2 single chain protein of *Artemia salina* it is a protein involved in membrane integrity in this organism by disruption of membrane, the zinc nanoparticles causes high mortality as shown by wet lab, also insilico study shows that at higher concentration of ZnO‐NPs these particles become binds strongly with the important protein of this specie *Artemia salina* and thus causes mortality, and hatching delay, as shown in the figure different strong interaction can be seen between ZnO‐NPs and Na+,K+‐ATPase alpha2 single chain protein of *Artemia salina*, these interaction includes positive–positive interaction, conventional bonding and strong H bonding among ligand and protein. Also binding energies this docked complex were shown in the Table 3.

**TABLE 3:** Docking score of Protein 8K1l with Lignad ZnONPs, docking by PyRx.

Ligand	Binding Affinity	rmsd/ub	rmsd/lb
8K1l_CB_ZnONPs_uff_E = 61.10	−5.1	0	0
8K1l_CB_ZnONPs_uff_E = 61.10	−5.1	40.18	37.329
8K1l_CB_ZnONPs_uff_E = 61.10	−5.1	40.185	37.32
8K1l_CB_ZnONPs_uff_E = 61.10	−5	16.93	13.925
8K1l_CB_ZnONPs_uff_E = 61.10	−5	17.159	14.067
8K1l_CB_ZnONPs_uff_E = 61.10	−4.9	11.386	8.765
8K1l_CB_ZnONPs_uff_E = 61.10	−4.9	11.718	8.773
8K1l_CB_ZnONPs_uff_E = 61.10	−4.9	10.945	8.753
8K1l_CB_ZnONPs_uff_E = 61.10	−4.4	4.314	2.524

## 4 Discussion

The present work successfully synthesized ZnO nanoparticles (ZnO‐NPs) using blue ‐green algae (Cyanophyte) as a natural precursor, which demonstrates a route for ecofriendly synthesis of ZnO‐NPs. FTIR analysis confirmed an efficient procedure for the synthesis of ZnO nanoparticles (ZnO‐NPs) using blue‐green algae. The presence of the characteristic peak at 632 cm^−1^ confirmed the wurtzite crystal structure of ZnO NPs which corroborates with Nilavukkarasi et al. (2020), Manohar et al. (2021). Hydroxyl group (1624 cm^−1^) and carbonate residues (1413 and 1329 cm^−1^) peaks indicated the contribution of algal metabolites towards reduction and stabilization of Zn ions in accordance with Faisal et al. (2021) and Bayrami et al. (2018). As dual functionalities, metabolites facilitate nanoparticle formation and functionalize their surfaces, making them more biocompatible.

Sharp intense diffraction peaks distinct to the hexagonal wurtzite structure were observed, which led to the conclusion that the ZnO NPs are highly crystalline and phase pure as observed from XRD peaks. The results of this study agreed with the ones of Chandra et al. (2019) and Saha et al. (2018), as high crystallinity was seen as essential to the functional performance of the ZnO‐NPs. The lack of impurity peaks further demonstrates the efficiency of algal mediated synthesis method. Meanwhile^,^ [31] also reported similar purity levels for ZnO NPs obtained using *Padina pavonica* extract cell free extracts, signifying the reliability of the biosynthetic approaches.

SEM and TEM analysis revealed morphological fine structure and a ZnO‐NP size distribution ranging from 20 to 90 nm. According to these findings, these biologically produced ZnO NPs produced the same sizes and morphologies reported in previous studies, like [32] and [33]. Uniformity and minimal aggregation in SEM and TEM images point to strong interparticle interactions stabilized by algal metabolites as seen in the results of [34]. Such a uniformity makes nanoparticles more bioactive and consistent in biomedical applications.

Among all tested material ZnO NPs exhibited excellent antibacterial efficacy, as the sizes of inhibition zones against *K. pneumoniae* and *E. coli* and *S. aureus* were bigger than that caused by the conventional antibiotics. Several other studies showed that ZnO NPs had a comparable antibacterial effect as did [34] and [35] who reported better results than standard antibiotics against bacterial pathogens. The dual mechanism of action of [36] via ROS generation and membrane destabilization finds support in the findings.

Further to this, time kill assays verified the concentration and time dependent antibacterial activity of ZnO‐NPs. Exposure resulted in significant reductions in bacterial density within certain time of exposure, with *E. coli* the most sensitive. The results obtained here are in line with those of [37] who attributed the time dependent bacterial inhibition by ZnO NPs to the ROS generating capability [38]. This study reveals the rapid bactericidal action and, therefore, the potential of ZnO NPs for acute infections management.

ZnO NPs showed antibiofilm activity against tested bacterial strains by inhibiting and eradicating biofilms synthesized. At higher concentrations (100 µg/mL), we observed up to 95% reduction in *S. aureus* biofilm biomass with highest sensitivity [39]. observed extensive biofilm inhibition and removal by ZnO‐NPs, which agrees with this study. Plant based phytochemical can tailor Zn)‐NPs properties to optimize antimicrobial performance, offering potential for environmentally friendly applications in biomedical and antimicrobial formulations [40]. The strong biofilm inhibition observed against *S. aureus* and *K. pneumoniae* may be from ZnO and phyto‐derived synergistic effetcs. ZnO NPs can disrupt the biofilm structure and therefore can provide a good solution in the sensor to treat biofilm associated infections. Our docking study yielded distinctive results, with a strong correlation between the wet lab and docking validation. The PBP2a exhibited a binding score of −7.7, indicating that ZnONPs significantly inhibited the PBP2a protein of *S. aureus*, a finding corroborated by the wet lab study, which produced analogous results.

A dose dependent effect of ZnO NPs towards Artemia salina was observed in cytotoxicity assessment; ZnO NPs were nontoxic at lower concentrations (5–10 µg/mL) [41]. Found similar biocompatibility of ZnO NPs at low concentration and toxicity at high amount. Determination of safe and effective concentrations for biomedical applications are based on this dose‐dependent cytotoxicity. Zinc nanoparticles induce significant mortality, as demonstrated by wet lab studies. Additionally, in silico analyses reveal that at elevated concentrations of ZnO‐NPs, these particles exhibit considerable binding affinity to critical membrane proteins in the species *Artemia salina*, resulting in increased mortality and hatching delays.

Also the observed antioxidant activity of ZnO nanoparticles is consistent with reported literature values of moderate to significant radical scavenging potential for ZnO nanoparticles. For instance, a similar concentration‐dependent increase in DPPH radical scavenging activity was reported by [12] but they used different extract for nanoparticles synthesis. The antioxidant activity of pristine ZnO nanoparticles is moderate compared to other conventional antioxidants such as ascorbic acid. Nevertheless, their reduced activity can be advantageous with respect to released applications in which sustained and controlled antioxidant release is desired while small molecules such as ascorbic acid act quickly.

The use of blue‐green algae for ZnO‐NP synthesis is compared to conventional synthesis methods where such use has benefits in the areas of sustainability, cost, and environmental safety. The FTIR analysis showed the algal metabolites also functioned as capping agents and facilitated reduction of Zn ions. These results agree with observations by [42] that biological precursors can increase the stability and functionality of nanoparticles.

## 5 Conclusion

This study represents a sustainable synthesis of zinc nanoparticles using blue green algae *Spirulina platensis* yielding a crystalline and pure ZnONPs with uniform shape and morphology. This particles had shown strong antibiofilm and antimicrobial activities, with efficacy of suppressing some standard antibiotics and also validated through docking and wet lab studies against PBP2a of *S. aureus*. Although the toxicity were only assessed *in Artemia salina* results suggest dose dependent effects, highlighting for further mammalian validation and also invivo. Overall the whole findings indicate the potential of ZnO‐NPs as ecofriendly agents, contributing to the advancement of green nanotechnology with promising biomedical and environmental application.

## Conflicts of Interest

The authors declare no conflicts of interest.

## Data Availability

Data sharing is not applicable to this article as no new data were created or analyzed in this study.
